# An Integrated Experimental Design for the Assessment of Multiple Toxicological End Points in Rat Bioassays

**DOI:** 10.1289/EHP419

**Published:** 2016-07-22

**Authors:** Fabiana Manservisi, Clara Babot Marquillas, Annalisa Buscaroli, James Huff, Michelina Lauriola, Daniele Mandrioli, Marco Manservigi, Simona Panzacchi, Ellen K. Silbergeld, Fiorella Belpoggi

**Affiliations:** 1 Cesare Maltoni Cancer Research Center, Ramazzini Institute, Bentivoglio, Bologna, Italy; 2 Leonardo da Vinci Programme at the Cesare Maltoni Cancer Research Center, Ramazzini Institute, Bentivoglio, Bologna, Italy; 3 National Institute of Environmental Health Sciences, National Institutes of Health, Department of Health and Human Services, Research Triangle Park, North Carolina, USA; 4 Johns Hopkins Bloomberg School of Public Health, Baltimore, Maryland, USA

## Abstract

**Background:**

For nearly five decades long-term studies in rodents have been the accepted benchmark for assessing chronic long-term toxic effects, particularly carcinogenicity, of chemicals. The European Food Safety Authority (EFSA) and the World Health Organization (WHO) have pointed out that the current set of internationally utilized test methods capture only some of the potential adverse effects associated with exposures to these agents over the lifetime.

**Objectives:**

In this paper, we propose the adaption of the carcinogenicity bioassay to integrate additional protocols for comprehensive long-term toxicity assessment that includes developmental exposures and long-term outcomes, capable of generating information on a broad spectrum of different end points.

**Discussion:**

An integrated study design based on a stepwise process is described that includes the priority end points of the Economic Co-operation and Development and the National Toxicology Program guidelines on carcinogenicity and chronic toxicity and developmental and reproductive toxicity. Integrating a comprehensive set of relevant toxicological end points in a single protocol represents an opportunity to optimize animal use in accordance with the 3Rs (replacement, reduction and refinement). This strategy has the potential to provide sufficient data on multiple windows of susceptibility of specific interest for risk assessments and public health decision-making by including prenatal, lactational, neonatal exposures and evaluating outcomes over the lifespan.

**Conclusion:**

This integrated study design is efficient in that the same generational cohort of rats used for evaluating long-term outcomes can be monitored in satellite parallel experiments to measure biomarkers and other parameters related to system-specific responses including metabolic alterations and endocrine disturbances.

**Citation:**

Manservisi F, Babot Marquillas C, Buscaroli A, Huff J, Lauriola M, Mandrioli D, Manservigi M, Panzacchi S, Silbergeld EK, Belpoggi F. 2017. An integrated experimental design for the assessment of multiple toxicological end points in rat bioassays. Environ Health Perspect 125:289–295; http://dx.doi.org/10.1289/EHP419

## Introduction

Synthetic chemicals have been used individually and as mixtures in consumer products for over a century, gaining intense momentum beginning after World War II. Naturally occurring elements and compounds have been used for millennia. The first bioassays for identifying chemicals posing a greater and immediate danger for carcinogenicity to individuals were first developed about 100 years ago (Yamagiwa and Ichikawa 1918). The chemical carcinogenesis revolution and testing age began when Yamagiwa and Ichikawa in 1918 showed that coal tar applied to rabbit ears caused skin carcinomas (Yamagiwa and Ichikawa 1918). The real impetus for testing chemicals came with passage of legislation, first in the United States in 1976 and then in several European Union member states, requiring evaluation of industrial chemicals, especially those in the workplace and in consumer products. This led to the development of a multi-national effort to harmonize testing methods through the Environment Programme of the Organization for Economic Co-operation and Development (OECD). Over the last 30 years, many test guidelines were developed within the OECD as well as the concepts for assessing risks of chemicals identified as harmful and carcinogenic in the workplace and environment (Hartung 2009; Huff 1992; Maltoni 1976; Soffritti et al. 2002; Tomatis 1979; Silbergeld et al. 2015). Rodent bioassays have been described in the OECD Test Guideline (TG) 453 (OECD 2009) and by the U.S. National Toxicology Program (NTP 2011b), with specifications for design and conduct of studies to evaluate toxic and carcinogenic potential of chemical, biological and physical agents in laboratory animals. Recognizing that carcinogenesis is a multi-step, multivariate process (Brash and Cairns 2009; Hanahan and Weinberg 2011), it may be unrealistic to expect a basic 2-year cancer study to provide all the complex data necessary for cancer risk identification, management, and regulatory decisions. Current OECD guidelines (OECD 2009), as planned, are not aimed to monitor cancer hazards and risks of exposure on susceptible individuals such as children and the elderly. For some test articles, NTP carcinogenicity 2-year protocol might include perinatal exposure, but these are selected only after considering patterns of human exposure (NTP 2011b, 2016). Furthermore, traditional toxicity testing methods could not identify many of the endocrine-related adverse effects of some chemicals, especially subtle effects on specific developmental stages (Bergman et al. 2012, 2015; Birnbaum 2013; Huff 1996; Huff et al. 1996; Manservisi et al. 2015; Melnick et al. 2002; Vandenberg et al. 2012), as happened for bisphenol A (Maffini et al. 2006; Vandenberg et al. 2009; vom Saal et al. 2007). Consistent with these considerations, both OECD and NTP have introduced new guidelines for reproductive and developmental toxicity with more functional end points to assess how agents affect the reproductive and endocrine status of animals (NTP 2011a; OECD 2011).

Study designs and outcomes investigated by current guidelines and our proposed protocol on carcinogenicity and chronic toxicity and reproductive and developmental toxicity are summarized in Table 1. The OECD reference guideline for reproductive and developmental toxicity, OECD TG 443 (Extended One-Generation Reproductive Toxicity Study), provides an evaluation of reproductive and developmental effects that may occur in offspring as a result of pre- and post-natal chemical exposure as well as systemic toxicity in pregnant and lactating females (OECD 2011). In the OECD TG 443 protocol, sexually mature male and female rodents [parental (P) generation] are exposed to graduated doses of test substances starting 2 weeks before mating and continued through mating, gestation, lactation, and weaning of pups (F1 generation). At weaning, pups are assigned to three groups for reproductive and developmental toxicity testing (cohort 1), developmental neurotoxicity testing (cohort 2), and developmental immunotoxicity testing (cohort 3). Other F1 offspring are exposed after weaning through adulthood. Clinical observations and pathology examinations are performed on all animals for signs of toxicity, with special emphasis on integrity and performance of male and female reproductive systems and health, growth, development, and function of offspring. Part of cohort 1 (cohort 1B) may be extended to include an F2 generation: In this case, procedures for F1 animals are similar to those for the P animals. The total number of animals involved in this OECD protocol design is more than one thousand (OECD 2011).

**Table 1:** Comparison between existing NTP MOG and OECD guidelines and the Ramazzini Institute (RI) proposed study design.

Study protocol	Number of animals	Issue												
		WOS/cohort	Start of treatment	End of treatment (weeks)	Age at necropsy (weeks)	Generation	DRF	Chronic toxicity/ carcinogenicity	Sub-chronic toxicity	Reprod/ develop	Neuro-toxicity	Neuro-behavioral	Immuno-toxicity	Teratology
OECD TG 453	480	Chronic toxicity/carcinogenicity	6–8 weeks	104	108	F1	X	X	X	—	—	—	—	—
OECD TG 443	1,760^*a*^	Reproductive (1A)	2 weeks PB	13	13	F0, F1	X	—	—	X	X	X	X	—
		Reproductive (1B)	2 weeks PB	14 or 20–25 if triggered^*a*^	14 or 20–25 if triggered^*a*^	F0, F1, F2 if triggered^*a*^
		Neurobehavioral (2A)	2 weeks PB	11–12	11–12	F0, F1
		Neurotoxicity (2B)	2 weeks PB	3	3	F0, F1
		Immunotoxicity (3)	2 weeks PB	8	8	F0, F1
NTP MOG	3,200^*a*^	Reproduction	GD 6	22	22	F0, F1, F2	X	—	X	X	X	X	X	X
		Prenatal/teratology	GD 6	18	18	F1, F2								
		13-week	GD 6	18	18	F1								
		Developmental/neurotoxicity	GD 6	11	11	F1								
		Developmental/immunotoxicity	GD 6	8	8	F1								
Total animals: 2,240–3,680 (OECD 453 and OECD 443 or NTP MOG)														
RI	1,720^*a*^	Chronic toxicity/carcinogenicity	GD 12	104	130 (final sacrifice)	F1	X	X	X	X	X	X	X	—
		Prenatal	Mating	Birth	3	F0, F1								
		Postnatal	PND 1	3	3	F0, F1								
		Prepubertal	3 weeks	6	6	F0, F1								
		Pubertal	6 weeks	9	9	F1								
		Adulthood	PND 1	26	26	F0, F1, F2								
Note: —, end point not covered in the study protocol; DRF, dose range finding; F0, parental animals; F1, litters generated by F0 animals; F2, litters generated by F1 animals; GD, gestation day; PB, prebreed; PND, postnatal day; X, end point covered in the study protocol. ^***a***^Considering 15 pups/litter in F2 generation (OECD TG 443 generates F2 only if triggered, while NTP MOG and RI include F2 generation by default).														

The NTP reference guideline for reproductive and developmental toxicity, the NTP’s Modified One-Generation (MOG) Reproduction Study (NTP 2011a), employs pregnant animals with exposures beginning at implantation with continued dosing of dams throughout gestation and lactation (Foster 2014). At weaning, offspring are administered the test substance at the same level as their respective dams and are assigned to different cohorts: a prechronic toxicity cohort (analogous to a standard 90-day study) for evaluating clinical pathology and target organ toxicity and pathology; a teratology cohort for evaluating prenatal development; another cohort to evaluate breeding and littering for potential examination of the subsequent generation. This study design involves exposure of pregnant females throughout gestation (the P generation), lifetime exposure of the F1, and generation of two cohorts of F2 animals (developmental and reproductive).

The OECD TG 443 and the NTP MOG were introduced only recently, and there is still no published data comparing studies with the same substance according to the two guidelines. We cannot exclude the possibility that authorities such as the U.S. Environmental Protection Agency (EPA), the U.S. Food and Drug Administration (FDA), the European Chemical Agency (ECHA), and the European Food Safety Agency (EFSA) could require (or have already required) the repetition of the tests with both guidelines considering the need for empirical evidence supporting the use of one of the two. It is our opinion that regularly studying the same substance with both the NTP MOG and OECD TG 443 represents an unnecessary repetition. The NTP’s MOG is able to generate large and robust data sets that include early-life exposure and teratogenicity, but requires a larger number of animals than the OECD TG 443 (Schiffelers et al. 2015; Foster 2014).

Starting from the 1990s, the Cesare Maltoni Cancer Research Centre (CMCRC) of the Ramazzini Institute (RI) performed carcinogenicity studies on low doses of chemical or physical agents that may expose millions or even billions of people to potential carcinogenic risks, such as radiations and food additives (Maltoni et al. 1985, 1999; Soffritti et al. 1999, 2002, 2007, 2008), using an alternative model, more sensitive than the traditional combined carcinogenicity and chronic toxicity 2-year protocol adopted by OECD and NTP (Bucher 2002; Huff 1992; Melnick et al. 2008). The CMCRC protocol includes prolonged periods of exposure and observation of experimental animals and starting exposures from the 12th day of fetal life (gestation) and continuing through lactation and weaning until at least 130 weeks or longer (Soffritti et al. 2002). In fact, human exposures to environmental agents, also at relatively low doses, most often starts prior to and during mother’s gestation, continues through lactation (via breast milk) and lasts until death. In standard bioassays, exposure generally starts in young adulthood and lasts until about 2 years, which is roughly equivalent to only 65 years in humans (Maltoni et al. 1997; Haseman et al. 2001; Huff et al. 2008; Melnick et al. 2008). Group sizes in carcinogenicity studies should also be increased whenever required for sufficient statistical power and to avoid the possibility of false negative response: Bioassays involving 100 animals or more per sex per group might be necessary for identifying carcinogenic effects of low doses and weak carcinogenic activity (Maltoni et al. 1981; McCormick 2013). More than 500 chemical-specific bioassays have been performed at CMCRC, and the results are used worldwide for hazard identification and human cancer risk assessments (NRC 2014a, 2014b).

To satisfy the need to consider multiple effects (e.g., cancer and noncancer) across multiple life stages and to reduce the overall number of animals required for separate studies of these end points, we propose the following experimental design that integrates traditional cancer guidelines with more recent proposals of OECD and NTP for studying reproductive and developmental toxicity. This new integrated experimental design aims to maximize the end points measured for each animal, thus reducing the overall number of animals produced and utilized, in accordance with the 3Rs (replacement, reduction and refinement) (European Union 2010).

**Figure 1:**
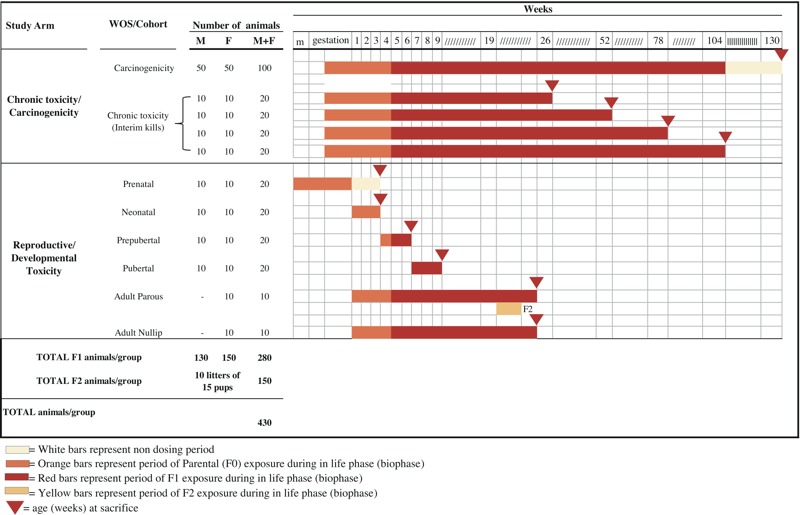
Integrated Long-Term Toxicity and Carcinogenicity Study experimental design. Schedule for treatment and duration for each group. Note: ////, continuous treatment; IIII, no treatment (period without dosing); F2, second generation offspring; m, mating; total animals/group, studying at least three exposure groups plus controls, the number for a comprehensive human equivalent hazard identification study is 1,720 animals; WOS, windows of susceptibility.

The central aim of the methodology proposed in the Integrated Long-Term Toxicity and Carcinogenicity Study is to maximize the breadth of outcomes assessed and to increase the sensitivity of testing beyond that in commonly used protocols to give more reliable and inclusive information on many important end points (Figure 1).

### Our Proposal: An Integrated Experimental Design

The integrated experimental design proposed by the CMCRC/RI is outlined in Figure 1 and more details on each specific section of the protocol are available in the Supplemental Material, “Ramazzini Institute’s proposal for Integrated Long-Term Toxicity/Carcinogenicity Study.” The study design is largely based on OECD TG 453 (modified only for duration of the experiment), OECD TG 443, NTP Guidelines. The study comprises the following components:

***Carcinogenicity and chronic toxicity study.*** Animals are treated from fetal life (dams, 12th day of pregnancy) until 104 weeks of age, then observed (with or without continuous exposure, depending on chemical) until 130 weeks of age (30 months). Interim kills are included to provide information on progression of non-neoplastic or neoplastic changes and mechanistic information (e.g., gene expression, serum biomarkers of inflammation, cell proliferation). Animals included for interim evaluation are also exposed from fetal life (dams, 12th day of pregnancy) until 26, 52, 78, and 104 weeks of age following OECD guidelines (OECD 2009).

***Reproductive and developmental toxicity.*** Different windows of susceptibility (WOS) related to reproductive and developmental and other noncancer effects are studied. The possible adverse effects of the substances are studied in prenatal, neonatal, prepubertal, pubertal, and adult parous and nulliparous WOS and compared among them, or with the possible long-term carcinogenicity effect.

### Animal Model

The laboratory rat has served as the traditional animal model of choice for research and regulatory developmental and reproductive toxicity testing conducted to support human health hazard identification and risk assessment. The rat has been used extensively for developmental and reproductive physiology and endocrinology research and has been more thoroughly characterized in these research fields than other species, likewise for identifying likely human carcinogens (Gray et al. 2004; Maltoni et al. 1999; Teitelbaum et al. 2015).

Our proposal to use Sprague-Dawley (SD) rats is based on the evidence that they are adequately sensitive, have a long history of being used in research studies, and are also recommended by the OECD (2009, 2011) and the NTP (King-Herbert and Thayer 2006; King-Herbert et al. 2010) and are used by many universities and organizations (Manservisi et al. 2015). SD rats are known and accepted as a human-equivalent model for cancer (Teitelbaum et al. 2015; Soffritti et al. 2006). The proposed protocol uses SD rat strains that meet the requirement of the OECD 443 and 453 guidelines: “strains with low fecundity or a well-known high incidence of spontaneous developmental defects should not be used” (OECD 2011) and “using a strain of animal that has an acceptable survival rate for the long-term study” (OECD 2009).

There are known limits for this animal model for individual cancer end points. For example, SD rats represent an optimal model for breast cancer research (Teitelbaum et al. 2015), while the high prevalence of benign tumors of the pituitary gland and pheocromocytoma of the subrenal gland make SD rats an inappropriate model for tumors of these organs (Dinse et al. 2010).

### Numbers of Animals

There is widespread agreement that the relatively small numbers of animals used in most standard toxicity tests is a serious issue in terms of sensitivity and reliability. On the other hand, there are social and ethical concerns about the number of animals used in these tests (Hartung and Rovida 2009). Inadequate tests are a main driver of additional testing, such that it can be argued that utilizing robust methods, with increased numbers of animals per test, will reduce overall animal testing. Current guidelines recommend study designs which encompass at least three treatment groups plus control. For the OECD TG 453 carcinogenicity and chronic toxicity protocol the minimal number of animals is 480; for the OECD TG 443, the minimal number is 1,760 and for the NTP MOG Reproduction Study, it is 3,200 animals (Table 1). But because only a limited number of end points are assessed in each of these tests, more animals are expected to be required to empower a broad-based toxicological evaluation for hazard and risk assessment. Performing these studies separately, as is current practice, would require up to 3,680 animals (Table 1).

In our proposal, breeders (virgin males and females) of about 10–15 weeks of age are matched in a single outbred mating, in a number adequate to obtain sufficient animals for the study. The objective of breeding is to generate animals in order to have no more than one sister and one brother for each control and exposed group (two sisters and two brothers in the carcinogenicity arm) in order to avoid any bias due to familial relationship.

Studying at least three exposure groups plus controls, the number for a comprehensive human equivalent hazard identification study is 1,720 animals (Figure 1 and Table 1). A higher number of exposed and control animals included in the studies better guarantees higher sensitivity of the model, sufficient statistical power, and overall saving animals that would be sacrificed in unnecessary repetition of the studies or performing uninformative underpowered studies (Hooijmans et al. 2010).

In compliance with the 3Rs, we suggest, whenever possible, to avoid the use of culling and to use all the pups generated during the experiment, avoiding unnecessary sacrifice of animals. It is our opinion that avoiding culling also would permit generally a more rigorous measure of litter mortality and simulate a human equivalent scenario, with more genetic variability and avoiding possible selection bias (for example selecting only healthy animals with higher birth weight). Nevertheless, the use of culling might be appropriate for studying suspect endocrine disruptor substances, as litter size can impact the weights and the growth rate of the pups, which can affect the timing of puberty. Puberty timing regulates other end points, so that the change in body weight from not equalizing litter size early on might have an inadvertent impact on the study.

### Dose Ranges

Under current testing procedures (Maronpot et al. 2004), when toxicology studies are performed, relatively high doses of a chemical are given to animals, generally higher than the doses humans are exposed to. However this is not always the case, especially for various workplaces and occupations and high-dose drug and cancer chemotherapies. Toxicity testing is typically carried out with maximum tolerated dose (MTD), previously determined in shorter-term exposures experiments of 28–90 days. Toxicology studies of higher doses show that a chemical can be lethal (and needs to be avoided), or block or disrupt pregnancies, or induce birth defects. These high-dose effects may not always be observed at lower doses, which is why some assume that these are safe exposures, but there may be other end points affected, that cannot be detected by typical methods of a standard bioassay (Teitelbaum et al. 2015). Non-monotonic dose–response curves reveal such unexpected effects, especially for endocrine disrupting chemicals (EDCs) (e.g., plasticizers, pesticides, and other industrial chemicals) as shown by several toxicological and epidemiologic studies on noncancer end points that are relevant to metabolic disease (Thayer et al. 2005, 2006; Vandenberg et al. 2012). In the multitude of chemicals that have never been tested adequately at low doses but were already tested for carcinogenicity at high doses, we suggest testing doses in the range of actual highest human exposure, setting the LOAEL (lowest observed adverse effect level) from traditional toxicological studies as the highest dose, particularly in experiments designed to test endocrine-sensitive end points. For chemicals never tested for long-term carcinogenic effects, at least one high-dose group near the MTD should be included, obviating the problem of unnecessary repetition of the bioassay if the low dose protocol is not carcinogenic.

Estimation of daily intake of a test substance depends on knowledge of the toxicokinetics, including route of administration, distribution, metabolism, and excretion, which are not all readily available from the literature (Søeborg et al. 2014). If a range of doses is unavailable or unknown, we propose that a dose-range finding (DRF) should be performed before starting the experimental protocol in order to determine an optimal exposure concentration for each chemical selected as close as possible to the estimated human exposure; in particular, when novel food (European Commission 2017) or similar test compounds are studied, nutritional aspects and other relevant methodological aspects (e.g., bioavailability, food metabolism that might differ in rodents and humans, stability of the test compound) related to exposure should be studied (EFSA 2013). When conducting exposure studies with low doses [many orders of magnitude lower than the no observed adverse effect level (NOAEL)], a systematic dose-calibration study should be performed in an appropriate rodent model in order to identify the administered oral dose of the test substance that results in biomarker concentrations (e.g., urine, serum) comparable to the ones observed in human population (Teitelbaum et al. 2016). Of course other higher doses must be chosen to adequately challenge biological systems and to provide some observable indication of toxicity, without jeopardizing the health and well-being or the body weights and survival of exposed animals, as well as being optimally sensitive to adequately evaluate the potential carcinogenicity (Bucher 2000; Huff 1999; Melnick et al. 2008). Higher doses also increase *a priori* statistical power to detect noncancer effects using a relatively small number of animals, although remarkable exceptions exist particularly for endocrine effects (Vandenberg et al. 2012).

### Timing of Exposure

Adult exposure to some chemicals is certainly an important factor in adverse health outcomes; however, increased focus on the fetus and neonate is of primary concern since developing organisms are extremely sensitive to perturbations by chemicals, especially those with hormone-like activity. Certain types of adverse effects may be more severe in developing organisms and occur at chemical concentrations that are in some instances below levels that would be considered harmful in adults (Tabb and Blumberg 2006). Few guidelines for testing environmental chemicals include prenatal or early-life exposures, and thus often do not provide information on risks of carcinogens related to early-life exposure (Rudel et al. 2011; Tabb and Blumberg 2006). Based on results of long-term carcinogenicity bioassays testing chemical and physical agents using rodents, there is ample evidence demonstrating that exposures during early developmental phases produce an overall increase of malignant tumors and increases of specific organ site neoplasms related to exposures to specific carcinogens as in the case of vinyl chloride and benzene (Maltoni et al. 1981, 1989; Huff et al. 2008; Soffritti et al. 2008). Early exposure to chemicals is particularly important in study designs if there is reason to believe human exposures begin *in utero* and that susceptibility may be greater during growth and early developmental stages (Rice et al. 1989).

For a clear understanding of this protocol, it should be considered that 16 weeks of age in adult rats roughly correspond to 10 years of age in human years (Sengupta 2013). In our proposal, animals belonging to the chronic toxicity and carcinogenicity study arm are observed until 130 weeks of age (corresponding to about 75–80 years of age in humans), starting exposure during fetal life (dams, 12th day of pregnancy), whereas OECD guidelines stipulate that animals should be killed and examined at 104 weeks of treatment (corresponding to about 60–65 years of age in humans) (Huff et al. 2008). Interim kills are also planned following the OECD TG 453 to provide information on the progression of non-neoplastic events and neoplastic changes and mechanistic information.

The Reproductive and Developmental Toxicity Study arm mimics human exposure during critical windows of development, and includes *a*) prenatal (F1) animals treated during embryonic life and sacrificed at postnatal day (PND) 21; *b*) postnatal (F1) animals treated through lactation, starting from birth (PND 1) and sacrificed at PND 21; *c*) prepubertal (F1) animals treated from PND 21 to PND 42; *d*) pubertal (F1) animals treated from PND 42 to PND 63; and *e*) adult parous and nulliparous (F1) female animals treated from PND 1 through lactation, until PND 181 (Figure 1). At 10–15 weeks, the parous group rats are mated (outbred), and chemical treatment is continued through pregnancy, delivery of pups (F2), and lactation. At the time of sacrifice of parous rats on PND 181, F2 pups had completed weaning.

In order to verify or elucidate effects in second generation, F2 offspring generated from F1 adult parous female rats are examined and sacrificed on PND 28.

During necropsy, frozen target tissues (including blood) and organs, together with paraffin-embedded tissues, are stored for histopathology and molecular biology studies, EDCs effects, neurotoxicity, biochemical and biohematological changes (metabolism), and toxic and preneoplastic lesions.

### Additional End Points and Adverse Effects of the Test Compound

The aim of our integrated experimental design was to investigate all or a majority of possible health effects related to exposure to the studied agent and to minimizing the unnecessary use of experimental animals. Our design also avoids wasted time when doing sequential end point studies. End points assessed in traditional toxicology and carcinogenicity testing are food and water consumption, chemical exposure, weight loss and gain, clinical pathology, survival and mortality, changes in organ weight, preneoplastic and neoplastic diseases with histopathological analyses. However, many examined chemicals have shown to also cause complex effects in animals, affecting organ development and functional and behavioral changes (Vandenberg et al. 2012). To best evaluate these fundamental end points, we included in our protocol design several of the NTP MOG and OECD TG 443 end points for immunotoxicity, neurotoxicity, and developmental and reproductive toxicity. It should be noted that this protocol is easily scalable (e.g., additional groups can be added if appropriate, or specific arms can be amended if previously investigated) and simple changes are feasible and would permit to target specific end points or tissues (for example sperm aneuploidy) that are not described in this proposal.

## Discussion

In our proposed lifetime experimental design, we assess a range of adverse outcomes of interest using a relatively large population of animals (sufficient power), born at the same time after mating of outbred breeders, randomized and studied for dose-related effects, with the lowest possible risk of bias (blinding of assessors of outcomes, randomization, blinded assessment of pathological lesions by a minimum of two assessors). Typically, for studying all the previously mentioned parameters (WOS, fertility, development, toxicity, carcinogenicity), approximately 10–20 studies are performed, using more animals, in different laboratories, with different procedures. Our experimental model and design overcomes these deficiencies and permits more information to be gathered on toxic, mechanistic, and biological parameters, using the same but fewer overall animals in a large but unique experiment. In fact, in our experimental design, rats from the same generation are used for studying chronic toxicity and carcinogenicity outcomes and distributed in satellite parallel experiments (WOS), thus minimizing variables between different arms of the multi-end point investigation, for detecting also reproductive/developmental toxicity.

Our integrated experimental protocol requires 1,720 animals, with a reduction up to 53% in animal use as compared to using separate test protocols (Table 1), representing an opportunity for investigating multiple toxicological end points at once, sparing animal lives in accordance with the 3Rs. We also expected an important reduction in terms of time, because the realization of a single integrated experiment would take a shorter time for design, approval, performance, and analysis if compared with multiple and sequential ones, which, in turn, would reduce costs and improve the availability of data for risk assessment.

The protocol we suggest addresses several important issues in the application of toxicological research to human health risk assessment including information on different toxicological outcomes of exposures and health hazards of importance to human populations that are currently not completely covered by standard test protocols; earlier initiation and longer duration of exposure and observation of animals (130 weeks of age instead of 110) for a more comprehensive analysis of potential effects of chemical exposures and outcome assessment; enabling interim analyses and other strategies to examine specific outcomes over the lifespan. For increased efficiency, results of these tests can be shared among laboratories. Ideally the *in vivo* biophase should be the responsibility of one laboratory in order to favor consistency of methods and quality of long-term animal studies (Gift et al. 2013). After the biophase, various end points, parameters, findings, and information on each category might be evaluated by different topic-expert scientists and laboratories. Frozen tissue samples from target organs are stored in order to study mechanistic aspects of the toxic process. Other relevant evidence, including cellular and molecular analyses related to mechanisms can be included in experimental designs, as has been proposed for the forthcoming OECD and NTP integrated guidelines regarding long-term *in vivo* studies (Darzynkiewicz et al. 2011; Kissling et al. 2007; Recio et al. 2010; Witt et al. 2008).

## Conclusions

This protocol represents a proposal to regulatory scientists and the scientific community in general.

Compared to other OECD and NTP guidelines, this protocol has the unique feature of integrating carcinogenicity, toxicity and reproductive and developmental toxicity end points in a single protocol, with animals of the same generation, exploring windows of susceptibility that are currently not addressed in the other guidelines design. The design and protocol discussed here requires validation in order to demonstrate that the combined test is feasible and is at least as good as the separate tests (OECD 2005). Experience in the application of this proposal will be required in order to reach the same level of confidence that has been achieved for the standard carcinogenicity bioassays (Huff 2010). *A priori* establishment of criteria and consensus on relevant end points of interest is also a good starting point for evidence-based evaluations and following systematic review of obtained results (Birnbaum et al. 2013; Mandrioli and Silbergeld 2016). This is clearly needed, for example, for testing endocrine-active substances with multiple end points, as well as modes and mechanisms of action, as the most reliably predictive animal model has yet to be identified. With this protocol, we aim to produce robust data sets that could also support the validation and discrimination of consensus criteria for evaluating noncancer outcomes, such as endocrine disruption.

We propose that conducting such integrated bioassays could enhance and expand scientific evidence for risk assessments, gathering sufficient and rapid information on several adverse effects in a unique protocol for protecting public health (Robinson 2012).

## Supplemental Material

(451 KB) PDF
