# Supplementary material for: An Integrated Experimental Design for the Assessment of Multiple Toxicological End Points in Rat Bioassays
Source: Environ Health Perspect. 2016 Jul 22;125(3):289–95. doi: 10.1289/EHP419 (PMC5332192; doi:10.1289/EHP419)
Supplement: (451 KB) PDF [file EHP419.s001.acco.pdf]

*Environ Health Perspect* DOI: 10.1289/EHP419
Advance Publication: Not Copyedited

**Note to readers with disabilities:** *EHP* strives to ensure that all journal content is accessible to all readers. However, some figures and Supplemental Material published in *EHP* articles may not conform to 508 standards due to the complexity of the information being presented. If you need assistance accessing journal content, please contact ehp508@niehs.nih.gov. Our staff will work with you to assess and meet your accessibility needs within 3 working days.

# Supplemental Material

# An Integrated Experimental Design for the Assessment of Multiple Toxicological End Points in Rat Bioassays

Fabiana Manservisi, Clara Babot Marquillas, Annalisa Buscaroli, James Huff, Michelina Lauriola, Daniele Mandrioli, Marco Manservigi, Simona Panzacchi, Ellen K. Silbergeld, and Fiorella Belpoggi

## Table of Contents

Ramazzini Institute's proposal for Integrated Long-Term Toxicity/Carcinogenicity Study.

Ramazzini Institute's proposal for Integrated Long-Term Toxicity/Carcinogenicity Study.

| ISSUE | SOURCE MATERIAL |
|---|---|
| **INITIAL CONSIDERATIONS AND OBJECTIVES** | **RAMAZZINI INSTITUTE (RI)**<br><br>**Integrated Long-Term Toxicity/Carcinogenicity Study guidelines** |
| **Objective** | The proposed Integrated Long-Term Toxicity/Carcinogenicity Study seeks from a single, large *in vivo* experiment to assimilate all possible information on the biological parameters needed for an appropriate, scientific evidenced-based risk-assessment as to possible human health hazards involving exposure of complex inputs such as chemicals with hormone-like activity. |
| **Study structure** | The proposed Integrated Long-Term Toxicity/Carcinogenicity Study is composed of two interrelated parts:<br>1. Dose-Range Finding (DRF): setting MTD as the highest dose for substances never tested for their carcinogenicity and LOAEL as highest dose for substances already tested adequately for their carcinogenicity In particular, if epidemiological studies are available, the DRF should determine the optimal testing dose for each chemical comparable to or higher than human exposure and dose calibration might be required for doses below NOAEL.<br>2. Integrated Long-Term Toxicity/Carcinogenicty Study: the study design is largely based on OECD TG 453[1] (modified only for duration of the experiment), OECD TG 443[2,3] and NTP Modified One Generation reproductive study[4,5] (MOG) guidelines. The study comprises two arms:<br>– Chronic toxicity/carcinogenicity: animals are treated from embryonic life until 104 weeks of age. Interim kills are included in the study design to provide information on progression of non-neoplastic changes and mechanistic information. The animals included for this purpose are treated from embryonic life until 26-52-78 and 104 weeks of age according to OECD TG 453.<br>– Reproductive/Developmental toxicity: satellite groups that mimic human exposure during critical windows of development (windows of susceptibility -WOS): prenatal, neonatal, prepubertal, pubertal, adult parous and nulliparous<br>The experimental design is showed in *Figure 3* |

| **TEST SUBSTANCE (TS)** | **RAMAZZINI INSTITUTE (RI)**<br><br>**Integrated Long-Term Toxicity/Carcinogenicity Study guidelines** |
|---|---|
| **Available information on the test substance**<br><br>*(OECD 443)* | Structure, molecular weight (MW), CAS#, source, alternative names, etc. Review of existing information: physico-chemical, toxicokinetic (TK), QSARs models, *in vitro* metabolic processes (to provide absorption, distribution, metabolism, and excretion –ADME- information), results of previous toxicity studies and relevant information on structural analogues. |
| **Consideration of toxicokinetic (TK) data**<br><br>*(OECD 443)* | TK data (ADME information) during the planning of the Integrated Long-Term Toxicity/Carcinogenicity study is strongly recommended. This information could aid informed decisions on selection of the route of administration, choice of vehicle, selection of doses, potential exposure of the offspring.<br>As suggested by OECD TG 443 the evaluation of pup exposure should be incorporated into the DRF designed to aid dose selection. Concentrations of test substance in pup blood and milk samples can be compared to maternal plasma levels at the same time point. These data would provide information on passage of the substance across the placenta, and/or lactational transfer and thus reveal information regarding exposure of both dams and pups. TG 443 states that TK data at the following time points from late pregnancy, mid-lactation and early post-weaning in dams and offspring would be very useful: 1) Late pregnancy (e.g. Gestation Day-GD 20) – maternal and fetal blood; 2) Mid-lactation (Post Natal Day-PND 10) – maternal and pup blood and/or milk; 3) Early post-weaning (e.g. PND 28) – weanling blood samples. |
| **Characterization of the test substance (TS)** | If the test substance is supplied, a representative sample from each dose of the initial formulated material preparation is analyzed to confirm concentration and homogeneity. A Preparatory Phase is necessary if the TS needs to be prepared: the objective is to assess all the procedures regarding test substance supply, analyses, stability, feed preparation. Samples of TS are evaluated for confirming of the presence of contaminants. Final concentrations of TS are confirmed in the different doses of each diet, according to standard protocols. |
| **Route of administration** | The routes used mimic human exposure. The routes most frequently used are: ingestion, injection, inhalation and external exposure (for physical agents, such as radiation). Rats are administered control- or test compound-containing feed ad libitum continuously (or water) containing the test article or dosed daily by gavage- include dose volume (e.g. 1 mL/adult animal and 0.5 mL/juvenile animal of 4-9 weeks of age). |
| **Choice of the vehicle**<br><br>*(OECD 443)* | The use of an aqueous solution/suspension is considered first, followed by consideration of a solution/suspension in oil (e.g. olive or corn oil). For vehicles other than water, the toxic characteristics of the vehicle should be known. The stability of the test substance in the vehicle should be determined. Considerations: effects on the absorption, distribution, metabolism or retention of the TS; effects on the chemical properties of the TS; effects on the food or water consumption or the nutritional status of the animals. |
| **Positive control** | If used (CAS #, Molecular formula, MW) |
| **Dose selection** | Based on preliminary DRF |

| **TEST ANIMALS** | **RAMAZZINI INSTITUTE (RI)**<br><br>**Integrated Long-Term Toxicity/Carcinogenicity Study guidelines** |
|---|---|
| **Selection of animal species and strain** | As suggested by both OECD TG 443, 453, and NTP Guidelines the rat is the preferred species. The strain of rat suggested is Sprague-Dawley (SD). Our proposal to use Sprague-Dawley (SD) rats is based on the evidence that they are extremely sensitive, currently being used in the NTP studies [6].<br>Rats from the same generation are used for studying chronic toxicity/carcinogenicity and reproductive/developmental toxicity, thus minimizing variables between different arms of the investigation. |
| **Age, body weight and inclusion criteria** | After the period of quarantine, breeders (male and female) of about 10-15 weeks of age are matched outbred in order to obtain the programmed experimental animals. Females should be nulliparous. The breeding facility has the objective to produce an adequate number of animals in order to build randomized dosed groups (no more than one sister and brother for each group). |
| **Housing** | Animals are housed as follows:<br>- Dams are housed one per cage by dose group.<br>- F1 pups shall remain with their respective dam until weaning on PND 28 (or euthanasia).<br>- After weaning, juveniles are group housed by litter and sex (up to 4 per cage as appropriate for their size). |
| **Diet and Water** | Diet: if present in the diet, TS content, homogeneity and stability should be verified. Samples of each batch of diet should be retained under appropriate conditions (e.g. frozen at -20 °C) until finalization of the report.<br>Tap water is administered to all other groups, changed and refilled daily.<br>Feed and drinking water are regularly analyzed for contaminants. |
| **Pre-study evaluation** | A sentinel animal program (SAP) is followed to ensure the health of the animals. The SAP is part of the periodic monitoring of animal health that occurs during the toxicological evaluation of different compounds/agents. Under this program the disease state of the rodent is monitored via serology on sera from extra (sentinel) animals in the studies rooms. These animals and the study animals are subject to identical environmental conditions. The sentinel animals come from the same production source and weanling groups as the animals used for the studies of the test material. |
| **Number and identification of animals**<br><br>*(OECD 443)* | Each F0 animal is assigned a unique identification number before dosing starts. All F1 offspring are uniquely identified when neonates are first examined on postnatal day (PND) 0 or 1. Records indicating the litter of origin should be maintained for all F1 animals, and F2 animals where applicable, throughout the study. |

| **PROCEDURES** | **RAMAZZINI INSTITUTE (RI)**<br><br>**Integrated Long-Term Toxicity/Carcinogenicity Study guidelines** |
|---|---|
| **Dosing F0 dams** | Chronic toxicity/carcinogenicity: from GD 12 to weaning<br>Reproductive/Developmental toxicity:<br>– Prenatal: from mating to delivery<br>– Neonatal: from delivery to PND21 of F1 offspring<br>– Prepubertal: from 3 weeks after delivery to weaning<br>– Pubertal: not treated<br>– Adult Parous and Nulliparous: from delivery to weaning |
| **Dosing F1 female and male rats** | Chronic toxicity/carcinogenicity: from GD 12 to 104 weeks. The F1 animals included in the interim kills are treated from GD 12 to 26, 52, 78, 104 weeks.<br>Reproductive/Developmental toxicity:<br>– Prenatal: pups are indirectly dosed via placenta, during embrionic life<br>– Neonatal: pups are indirectly dosed through lactation beginning at birth<br>– Prepubertal: pups are indirectly dosed through lactation for one week (PND 21-PND 28) and are dosed directly beginning at weaning on PND 28, continuosly until 6 weeks of age<br>– Pubertal: from 6 to 9 weeks of age<br>– Adult Parous and Nulliparous: pups are indirectly dosed through lactation beginning at birth and are dosed directly beginning at weaning on PND 28, continuosly until 26 weeks of age |
| **Dosing F2 offspring** | Female and male rats belonging to the F2 generation are continuously treated from their embryonic life to weaning (through breast milk). |
| **Mating** *(OECD 443)* | Each P female is placed with a single, randomly selected, unrelated male from the same dose group (1:1 pairing) until evidence of copulation is observed or 2 weeks have elapsed. Day 0 of pregnancy is defined as the day on which mating evidence is confirmed (a vaginal plug or sperm are found). |
| **Litter size** | After delivery, the newborns (F1 litters) are examined for the presence of live and dead pups as soon as possible. No pup culling takes place before weaning. All the F1 pups are housed with their dam until weaning, then they are separated, identified by ear punch and housed individually. Each litter contributed at least with 1 female and male pup. All exceeding pups are not included in the study. |
| **F1 disposition** | Chronic toxicity/carcinogenicity: F1 animals treated from embryonic life (GD 12) until 104 weeks of age and observed until final sacrifice (130 weeks). The F1 animals included in the interim kills are treated from GD 12 until 26-52-78 and 104 weeks of age then sacrificed.<br>Reproductive/Developmental toxicity:<br>– Prenatal: animals treated during their embrionic life and sacrificed at 3 weeks of age<br>– Postnatal: animals treated trough lactation, starting from birth and sacrificed at 3 weeks of age<br>– Pre-pubertal: animals treated from 3 to 6 weeks of age<br>– Pubertal: animals treated from 6 to 9 weeks of age<br>– Adult Parous: animals exposed starting at birth; females mated once adult; female will complete pregnancy, delivery and pups' weaning; female and male observed until 26 weeks of age<br>– Adult Nulliparous: animals exposed starting at birth and sacrificed at 26 weeks of age |
| **Second mating of the F1 animals** | In order to verify or elucidate an effect on the second generation, F1 adult parous female (from Reproductive/Developmental toxicity arm): rats are mated at 10-15 weeks of age in order to generate F2 offspring. On PND 28, F2 litters could be sacrificed. |

| **<u>IN-LIFE ENDPOINTS (part I)</u>** | **<u>RAMAZZINI INSTITUTE (RI)</u>**<br><br>**<u>Integrated Long-Term Toxicity/Carcinogenicity Study guidelines</u>** |
|---|---|
| **Clinical observations (P and F1 after weaning)**<br><br>*(OECD 443 + NTP MOG)* | Formal (out of cage) clinical observations once daily; cage side observations at least twice daily, including, moribundity and/or death. In the case of gavage dosing, the timing of clinical observations should be prior to and post dosing (for possible signs of toxicity associated with peak plasma concentration). Pertinent behavioural changes, signs of difficult or prolonged parturition and all signs of toxicity are recorded. |
| **Body weight**<br><br>*(NTP MOG)* | – All breeders F0 generation (and F1 if mated once adult) are weighted before matching.<br>– Dams' body weights is recorded daily during gestation and during Lactation Day-LD 1, 4, 7, 14, 21 and 28. All weights are reported as well as gestational/post-natal weights for the following intervals: GD 3-6, 6-9, 9-12, 12-15, 15-18, 18-21, and 6-21; LD 1-4, 4-7, 7-10, 10-13, 13-16, 16-19,19-21, 21-25, 25-28, and 1-28.<br>– All F1and F2 offspring in the different groups are weighted at PND 1, 4, 7, 10, 13, 16, 19, 21, 25 and 28 (by sex and by litter).<br>– All F1 animals are weighed individually at weaning (PND 28) and weekly until PND 91, then once every two weeks until sacrifice.<br>– All animals are weighed at sacrifice. |
| **Food/water consumption**<br><br>*(NTP MOG)* | Dam feed and water consumption is measured for GD 3-6, 6-9, 9-12, 12-15, 15-18, 18-21, 6-21, and LD 1-4, 4-7, 7-10, 10-13, 13-16, 16-19,19-21, 21-25, 25-28, and 1-28<br>Daily water and feed consumption are measured per cage and body weights individually, once a week for the first 13 weeks starting from 6 weeks of age, and every two weeks until 110 weeks of age. |
| **Examination of puberty onset**<br><br>*(OECD 443 + NTP MOG)* | – <u>Female</u>: to determine the onset of puberty, from PND 25 the vaginal opening of five female rats in each group with animal of this age is checked daily until each animal acquires this developmental landmark. Body weight on day of acquisition is registered. Starting 3 days after the onset of vaginal opening, daily vaginal lavage is conducted for 14 days to determine first estrus. The estrous cycle is identified under a microscope (×100) using a vaginal smear flushed with physiological saline. The ages and body weights at which first ovulation in the F1 offspring occurs is recorded.<br>– <u>Male</u>: from PND 35 the balano-preputial separation of five male rats in each group with animal of this age is checked daily until each animal acquires this developmental landmark. |
| **Mating and pregnancy**<br><br>*(OECD 443)* | Endpoints:<br>– dates of pairing, insemination and parturition. The day on which parturition occurs is lactation day 0 (LD 0) for the dam and postnatal day 0 (PND 0) for the offspring;<br>– pre-coital interval (pairing to insemination);<br>– duration of pregnancy (insemination to parturition) are calculatedprecoital interval and duration of pregnancy;<br>– signs of dystocia, abnormal nesting behaviour, nursing performance. |
| **Offspring parameters**<br><br>*(NTP MOG)* | – PND 0: Number of live and dead offspring (by litter, and total)<br>– PND 1: Number of live and dead offspring (by litter, and total); anogenital distance (AGD) and corresponding pup weight.<br>– PND 4: Number of live and dead pups, sex, weight (by litter, sex, and total).<br>– Presence and type of developmental anomalies in the pups (e.g. clinical observations, gross external malformations).<br>– Number and weight of pups on PND 4, 7, 10, 13, 16, 19, 21, 24, 28 (by pup, sex and litter).<br>– Males: Presence of areolae on PND 13, testicular descent (PND 14-30), balano-preputial separation ((BPS) PND 35 until acquisition);and body weight on day of acquisition.<br>– Females: day of vaginal opening (PND 21 until acquisition) and body weight on day of acquisition and estrous cyclicity for 14 days vaginal opening<br>– F1 (after weaning): daily out of cage clinical observations; cage-side observations, twice daily, body weights and cage feed consumption |

| **IN-LIFE ENDPOINTS (part II)** | **RAMAZZINI INSTITUTE (RI)**<br><br>**Integrated Long-Term Toxicity/Carcinogenicity Study guidelines** |
|---|---|
| **Assessment of neurobiological development**<br><br>*(OECD 443)* | **Neurobehavioral**<br>Neurobehavioral tests should be conducted post-weaning (e.g., 28±2 days) and for young adults (PND 60 and older):<br>– motor activity<br>– auditory startle habituation test are performed on the offspring<br>– learning and memory<br>**Neuropathology:**<br>At the termination of the study neuropathological analysis should be conducted. Brains are removed, weighed, and immersion-fixed in an appropriate aldehyde fixative. Multiple sections are examined from the brain to allow examination of olfactory bulbs, cerebral cortex, hippocampus, basal ganglia, thalamus, hypothalamus, mid-brain (thecum, tegmentum, and cerebral peduncles), brain-stem and cerebellum |
| **Assessment of potential developmental immunotoxicity**<br><br>**(OECD 443)** | *T-celi dependent antibody response* |
| **Follow-up assessment of potential reproductive toxicity** | Female rats belonging to the Adult Parous Group of the Reproductive/Developmental Toxicity Arm are treated until 26 weeks and bred at 10-15 weeks to obtain a F2 generation. On PND 28, F2 litters are sacrificed. |

| **TERMINAL ENDPOINTS** | **RAMAZZINI INSTITUTE (RI)**<br><br>**Integrated Long-Term Toxicity/Carcinogenicity Study guidelines** |
|---|---|
| **Clinical biochemistry / Haematology**<br><br>*(NTP MOG)* | The hematological parameters and clinical chemistries measurements follow the NTP MOG guideline. : erythrocyte count, hemoglobin concentration, hematocrit (Packed cell volume), mean corpuscular volume, mean corpuscular hemoglobin, mean corpuscular hemoglobin concentration, leukocyte count, leukocyte differential, reticulocyte count, platelet count, morphologic assessment erythrocytes, leukocytes and platelets.<br>Clinical chemistries: total Protein, albumin, urea Nitrogen (BUN), creatinine, alanine Aminotransferase (ALT), sorbitol dehydrogenase (SDH), alkaline Phosphatase (ALP), total Bile Acids, glucose, creatine Kinase (CK), cholesterol, triglycerides. |
| **Blood for micronuclei**<br><br>*(NTP MOG)* | Samples of blood (~200 µl) are collected in EDTA from rats at termination. Samples are to be refrigerated immediately after collection and remain refrigerated until blood micronuclei analyses. |
| **Urinalysis** *(OECD 443)* | Urinalysis are performed prior to termination and the following parameters evaluated: appearance, volume, osmolality or specific gravity, pH, protein, glucose, blood and blood cells, cell debris. Urine may also be collected to monitor excretion of test substance and/or metabolite(s). |
| **Sperm parameters**<br><br>*(OECD 443)* | At termination, testis and epididymis weights are recorded for all males. At least one testis and one epididymis are reserved for histopathological examination. The remaining epididymis is used for enumeration of cauda epididymis sperm reserves. In addition, sperm from the cauda epididymis (or vas deferens) is collected using methods that minimize damage for evaluation of sperm motility and morphology. |
| **Mammary Glands**<br><br>*(NTP MOG)* | Axillary (second) and inguinal (fourth) mammary glands are dissected and placed on a piece of cardboard for histopathological examination. Axillary mammary glands are fixed in alcohol 70% for 48 h. Inguinal mammary glands are both frozen in Chronic toxicity/carcinogenicity arm. For females rats belonging to all the Reproductive/Developmental toxicity arm, the fourth left inguinal mammary gland is frozen and the contralateral gland is whole mounted and stained with carmine, at PND 21 (3 weeks), PND42 (6 weeks), PND63 (9 weeks), and PND182(26 weeks) (terminal sacrifices) |
| **Necropsy and histopathology**<br><br>*(OECD 443)* | **F0 animals:** rats are necropsied after F1 weaning has been completed. Animals are subjected to external examination and uterine implantation scars enumerated. Gross lesions and representative control tissues are retained, processed to slides and examined histopathologically. Rats that do not appear to be pregnant and do not deliver are examined for the presence of implants (using potassium ferricyanide) and corpora lutea enumerated.<br>**F1and F2 animals:** each animal undergoes complete necropsy and histopathological evaluation of all organs following natural death. The list of organs and tissues collected during necropsy follows the OECD TG 443. |
| **Organ weight**<br><br>*(OECD 443 + NTP MOG)* | Organ weights are determined from all animals. Those organs to be weighed are: brain, pituitary, liver, spleen, thymus, lung, heart, kidneys, adrenal glands, testes, epididymides, seminal vesicles w/coagulating glands, prostate, uterus with cervix and ovaries. |
| **Power calculation** | For the power calculation of each end-point we refer to OECD TG 453, 443 and NTP MOG guidelines, that are referenced in parentesis in each section [1,2,3,4,5] |

| **REPORTING** | **RAMAZZINI INSTITUTE (RI)**<br><br>**Integrated Long-Term Toxicity/Carcinogenicity Study guidelines** |
|---|---|
| **Data presentation**<br><br>*(NTP MOG)* | Mean values and index of variability (i.e. SE, or SD) for all protocol are provided, including (but not limited to):<br>– Mean body weights, body weight gains, clinical signs, food and water consumption<br>– Male and female fecundity and fertility indices<br>– Mean litter size, mean body weight of live pups (by sex, and combined) and total number live/dead pups.<br>– Pup survival on PND 1-4, 4-7, 7-14, 14-21<br>– AGD (by sex, nested by litter and co-varied by body weight)<br>– Mean number of areolae and nipples (by litter)<br>– Results of external, visceral and skeletal fetal examinations<br>– Organ weights |
| **Evaluation of results**<br><br>*(NTP MOG)* | The evaluation includes:<br>– A summary of findings for mortality, body weight, food consumption, etc.<br>– Breeding and littering results as well as any patterns observed for these findings<br>– Results of study-specific evaluations as appropriate<br>– Pathology findings; if applicable, relate to potential changes in fertility or other reproductive outcomes. |
| **Test report**<br><br>*(NTP MOG)* | Summary data are included in the results section of the final study report as follows:<br>– Introduction<br>– Materials and Methods<br>– Results<br>– Discussion<br>– Appendices<br>Individual animal data are to be included as an appendix to the final study report. Data are organized by species, sex and treatment group. Notations of any observation and/or action taken to confirm or explain atypical data points are to be included |
| **Discussion of results**<br><br>*(NTP MOG)* | Discussion section of the study report shall include correlations between clinical laboratory findings and anatomic pathologic changes and/or clinical signs exhibited by the study animals. |
| **Interpretation of results**<br><br>*(NTP MOG)* | Historical control data are provided to enhance interpretation of study results.<br>– Provide a discussion of treatment-related findings, including biological significance of the data<br>– Describe any problems encountered that would impact interpretation of data<br>– Provide a brief summary of salient findings |

**References**


1. OECD Test No. 453: Combined Chronic Toxicity/Carcinogenicity Studies, 08 Sep 2009
2. OECD Test no. 443: Extended one-generation reproductive toxicity study: OECD Publishing), 2011.
3. GUIDANCE DOCUMENT SUPPORTING OECD TEST GUIDELINE 443 ON THE EXTENDED ONE GENERATION REPRODUCTIVE TOXICITY TEST Series on Testing and Assessment No. 151. Environment directorate joint meeting of the chemicals committee and the working party on chemicals, pesticides and biotechnology. OECD, 09-Jul-2013
4. NTP. Ntp's modified one-generation reproduction study. 2011a.
5. DRAFT Protocol outline for the Modified One-generation Study (MOG #) of TEST ARTICLE (CAS#, TEST ARTICLE #) in Harlan Sprague Dawley Rats Exposed Via Dosed feed. Available online at http://ntp.niehs.nih.gov/testing/types/
6. King-Herbert, A.P., R.C. Sills and J.R. Bucher (2010), "Commentary: Update on Animal Models for NTP Studies", Toxicol Pathol, 38, 180-181.
